# Non-Steroidal Anti-Inflammatory Drugs: An Overview of Cardiovascular Risks

**DOI:** 10.3390/ph3072146

**Published:** 2010-07-07

**Authors:** Inger L. Meek, Mart A.F.J. van de Laar, Harald E. Vonkeman

**Affiliations:** Department of Rheumatology and Clinical Immunology, Medisch Spectrum Twente and University of Twente, Ariensplein 1, 7500 KA Enschede, The Netherlands; E-Mails: i.meek@mst.nl (I.L.M.); m.vandeLaar@mst.nl (M.AFJ.L.)

**Keywords:** NSAIDs, aspirin, cardiovascular events

## Abstract

While aspirin may offer protection, other non-aspirin non-steroidal anti-inflammatory drugs (NSAIDs) can cause serious cardiovascular side effects and complications. This has led to a general "black box" warning for cardiovascular adverse events for NSAIDs. This review explores the different mechanisms underlying the protective effects of aspirin, the NSAID associated renovascular effects causing hypertension, edema and heart failure, the cardiovascular effects causing myocardial infarction and stroke, and the possible deleterious interaction between NSAIDs and aspirin.

## 1. Introduction

Nonsteroidal anti-inflammatory drugs (NSAIDs) are among the most frequently prescribed drugs in modern medicine. NSAIDs are very effective in the alleviation of pain, fever and inflammation, and millions of patients worldwide have found relief in their use since the discovery of the soothing properties of willow bark more than 3,500 years ago. Furthermore, aspirin, the archetype of the NSAID family, has become the cornerstone of secondary prevention of thrombotic cardiovascular events. NSAID use is however associated with several serious treatment side effects, with considerable associated morbidity and mortality. Many of these side effects may be prevented by careful consideration of the patient’s risk factors and by subsequent implementation of preventive strategies.

## 2. Methods

We searched Medline for English-language articles published up to 2010, using the keywords acetylsalicylic acid, aspirin, NSAIDs, cyclooxygenase-2, adverse effects, and cardiovascular. The abstracts were screened for relevance and the publications relating to aspirin and NSAIDs were obtained. Additional references were identified from the bibliographies of the retrieved reports and from review articles. Further sources of information were retrieved from the internet.

## 3. Results and Discussion

### 3.1. Prostaglandins and COX

Prostaglandins are the members of a group of lipid compounds derived enzymatically from fatty acids. They are rapidly metabolized, act locally and are involved in many processes that cause inflammation after injury or illness, regulate the constriction of the uterus, affect constriction and relaxation of blood vessels, and are involved in the aggregation of blood platelets. The first prostaglandins to be discovered were isolated independently from seminal fluid in 1935 by both the Swedish physiologist Ulf von Euler and the British pharmacologist M.W. Goldblatt, and were thought to be a prostatic secretion, as reflected by their naming [1,2]. 

Prostaglandins are found in most tissues and organs and are produced by all nucleated cells, except lymphocytes, from essential fatty acids: gamma-linolenic acid, arachidonic acid, and eicosapentaenoic acid. In the early 1960s both Swedish and Dutch scientists worked to elucidate the mechanisms underlying the production and actions of these compounds. It was found that in humans, arachidonic acid is mobilized from cell-membrane lycerophospholipids by phospholipase A2. The subsequent biotransformation of arachidonic acid is catalyzed by prostaglandin G2/H2 synthase, resulting in the sequential formation of prostaglandin G2 (PGG2) and prostaglandin H2 (PGH2) via the cyclooxygenase (COX) activities of the protein. Tissue-specific prostaglandin synthases convert PGH2 into other prostaglandins and thromboxane, having different functions in different tissues. For example, PGD2 is involved in sleep regulation and allergic reactions; PGF2 controls the contraction of the uterus and bronchoconstriction, and thromboxane A2 (TXA2) stimulates the constriction of blood vessels and induces platelet aggregation. Prostacyclin (PGI2) dilates blood vessels, inhibits platelet aggregation, and may protect against damage to the stomach lining; prostaglandin E2 (PGE2) is involved in pain, inflammation, and fever and also acts to prevent damage to the stomach [3,4]. 

In 1989 Phillip Needleman confirmed the suspicion of two distinct isoforms of COX, being regulated and acting in distinct manners [5]. COX-1 showed to be constitutionally present in low abundance in most human tissues, acting as a housekeeping enzyme by regulating normal physiological processes like the maintenance of gastric mucosal integrity, kidney function, and platelet aggregation, whereas COX-2 was undetectable in most tissues under normal physiological circumstances and was selectively upregulated after exposure to inflammatory mediators or trauma, causing subsequent inflammatory responses and mediation of pain. 

Both COX isozymes are membrane-associated proteins with a 3-dimensional structure of a long narrow channel ending in a hairpin bend, and internalize adjacent arachidonic acid which is released when membrane damage occurs [6,7]. Arachidonic acid is bound high within the COX enzyme and is biotransformed via PGG2 into PGH2, which is a subsequent substrate for other cell and tissue-specific terminal enzymes, such as PGI2 synthase which produces prostacyclin, thromboxane synthase which produces thromboxane, and glutathione S-transferase for the conversion to PGE2. 

#### 3.1.1. Aspirin’s Affinity to COX

Aspirin exerts its effects by non-competitive and irreversible acetylation of the COX enzyme, in which an acetyl group is covalently attached to a serine residue in the active site of the COX enzyme, rendering the COX enzyme permanently inaccessible for the biotransformation of arachidonic acid into PGH2 and thus effectively inhibiting subsequent prostaglandin production [8,9]. Nucleated cells, such as the cells of the gastric mucosa and inflammatory cells, are able to newly synthesize COX-1 and COX-2 and thus recover COX function and prostaglandin production despite inhibition by aspirin. Blood platelets on the other hand have no cellular nucleus and therefore lack the ability to newly synthesize COX. Furthermore, in blood platelets TXA2 production is entirely COX-1 dependent, which is why COX-1 binding of aspirin in blood platelets will permanently prevent the production of TXA2 and subsequently inhibit platelet aggregation for the duration of the platelets’ lifecycle, making aspirin a potent cardiovascular protective agent [10,11].

#### 3.1.2. Aspirin: Benefits and Risks

Nowadays, the main indication of aspirin is in the prevention of occlusive cardiovascular disease. A collaborative meta-analysis of individual participant data from 16 randomised trials (17,000 individuals at high average risk, 43,000 person-years, 3,306 serious vascular events) on the secondary prevention of cardiovascular events, comparing long term aspirin with placebo on the occurrence of a new myocardial infarction or stroke or vascular death, showed a statistically and clinically significant reduction in serious vascular events (6.7% with aspirin *vs.* 8.2% with placebo per year), with similar results in both men and women [12]. The risk reduction was found in all subgroups of ischemic events, at the cost of an excess in major gastro-intestinal and other major extra cranial bleeds (RR 2.69, CI 1.25–5.76, data available from five out of 16 trials). However, the absolute incidence of major bleeding events was much lower, approximately 0.15% per year, resulting in an overall balance in favour of aspirin. For the primary prevention of cardiovascular events, the authors came to different conclusions. When analysing the data of six primary prevention trials (95,000 individuals at low average risk, 660,000 person-years, 3,554 serious vascular events) a statistically significant 12% proportional reduction in serious vascular events was found (0.51% with aspirin *vs.* 0.57% in controls per year), mainly due to a reduction in non-fatal myocardial infarctions (0.18% *vs.* 0.23% per year), at the cost of an increase in haemorrhagic stroke (0.04% *vs.* 0.03% per year) and major gastrointestinal and extra cranial bleeds (0.10% *vs.* 0.07% per year) [12]. In their discussion the authors argue that in modern times the risk-reducing effects of aspirin might be reduced by half by the prescription of other risk reducing medications, such as statins, while the risks of bleeding would remain the same, which would render the net beneficial effect in the primary prevention of cardiovascular events in low risk patients negligible. 

Another meta-analysis, searching for adverse events of low-dose aspirin in 22 randomized placebo-controlled trials, found a relative risk of 2.07 for major gastrointestinal bleeding with aspirin, with an absolute annual increase of 0.12% [13]. With this low absolute risk increase, the number needed to treat with aspirin to cause one major gastrointestinal bleeding is 833. Strategies for the prevention of aspirin associated gastrointestinal bleeding should therefore be targeted at high-risk patients, such as patients with previous gastro-intestinal bleeding, age over 60 years, concomitant use of corticosteroids, non-aspirin NSAIDs, anticoagulants, other platelet inhibitors and serotonin reuptake inhibitors, infection with *Helicobacter pylori*, and co morbid conditions such as diabetes mellitus, heart failure, and rheumatoid arthritis [14,15]. 

By inhibiting gastric COX-1, aspirin may reduce mucosal blood flow, causing local ischemic injury. Aspirin may also impair specific prostaglandin-dependent defences, which protect the gastric mucosa, such as the thick bicarbonate-containing mucous layer lining the interior of the stomach, which buffers luminal gastric acid and thus protects the stomach wall. When these defences have been weakened by aspirin induced inhibition of gastrointestinal COX-1, a second wave of injury caused by luminal gastric acid may facilitate deeper ulceration, bleeding, and even perforation of the stomach wall [16] Strategies aimed at preventing aspirin and non-aspirin NSAID gastropathy either help to maintain the integrity of the stomach wall and mucous lining, such as the concomitant administration of prostaglandin analogues, or alternatively inhibit the secretion of gastric acid, such as concomitant histamine H2-receptor antagonists or proton-pump inhibitors (PPI). 

Studies on the prevention of recurrent gastrointestinal bleeding are all on PPI based strategies, either PPI *vs.* placebo or PPI *vs. H. pylori* eradication, colonisation with H Pylori being associated with an increased risk of recurrent ulcer bleeding. In one study, 123 *H. pylori*-positive patients who had developed bleeding ulcers with low-dose aspirin were treated with *H. pylori* eradication therapy and subsequently randomized to lansoprazole 30 mg daily or placebo in addition to aspirin 100 mg daily [17]. At 12 months follow-up, the rate of recurrent ulcer complications was 1.6% with lansoprazole and 14.8% with placebo. Another study compared the efficacy of either *H. pylori* eradication or concomitant PPI treatment for the secondary prevention of aspirin ulcer bleeding [18]. This study enrolled 400 *H. pylori-*positive patients, 250 with low-dose aspirin and 150 with NSAIDs, who had presented with ulcer bleeding. Only the data for the 250 aspirin users will be presented here. After endoscopically confirmed ulcer healing with omeprazole 20 mg daily for eight weeks or longer, patients were given aspirin 80 mg daily and then randomized to omeprazole 20 mg daily for six months or one week of *H. pylori* eradication therapy followed by placebo for six months. The probability of recurrent ulcer bleeding during the 6-month follow-up period was 1.9% for patients receiving eradication therapy and 0.9% for those treated with omeprazole [18]. From these and other studies we may therefore conclude that in high risk patients with previous aspirin associated gastrointestinal bleeding, PPI treatment offers significant reduction in the risk of recurrent bleeding. 

### 3.2. The Family of Non-Steroidal Anti-Inflammatory Drugs

In 1959 John Nicholson from the Boots Company had, in collaboration with Stuart Adams, synthesized a drug with analgesic, antipyretic, and anti-inflammatory properties similar to aspirin. The drug was named ibuprofen and was marketed in 1969 under the brand name Brufen, despite performing no better than placebo in an initial clinical trial among 18 rheumatoid arthritis patients [19,20]. Ibuprofen would, however, become the first in a long series of very successful non-aspirin NSAIDs. Nowadays, approximately 50 different NSAID preparations are available and, as a class, they are among the most commonly prescribed drugs worldwide. The main indications are mild to moderate pain of somatic origin. Due to their anti-inflammatory effect, NSAIDs may be especially effective in inflammatory diseases such as rheumatoid arthritis. NSAIDs may be grouped as salicylates (with as a prominent member aspirin itself), arylalkanoic acids (diclofenac, indomethacin, nabumetone, sulindac), 2-arylpropionic acids or profens (ibuprofen, flurbiprofen, ketoprofen, naproxen), *N*-arylanthranilic acids or fenamic acids (mefenamic acid, meclofenamic acid), pyrazolidine derivates (phenylbutazone), oxicams (piroxicam, meloxicam), sulfonanilides (nimesulide), and others. The efficacy of NSAIDs may vary by patient and by indication. In case of inefficacy, substitution by a NSAID from a different chemical class is a sensible therapeutic option [10]. 

As a group, NSAIDs are structurally diverse and differ in pharmacokinetic and pharmacodynamic properties, but ultimately they share the same mode of action. Like aspirin, non-aspirin NSAIDs inhibit the production of prostaglandins by blocking the COX enzyme, causing analgesic, antipyretic, and anti-inflammatory benefits, but at a risk for increased gastro intestinal bleeding [21]. However, aspirin and non-aspirin NSAIDs differ fundamentally in the way the COX enzyme is inhibited. As mentioned previously, aspirin permanently inhibits COX by non competitive and irreversible acetylation. Conversely, non-aspirin NSAIDs competitively and reversibly inhibit the COX enzyme during only part of their dosage interval. This distinction is exemplified by their differential effects on platelet aggregation. As mentioned previously, blood platelets, unlike for instance inflammatory cells, have no cellular nucleus and are therefore unable to newly synthesize COX. Aspirin as an irreversible inhibitor of COX function permanently prevents the production of TXA2 and therefore inhibits platelet aggregation for the duration of the platelets’ lifecycle, making aspirin a potent cardiovascular protective agent. Conversely, as a result of their competitive reversible binding of the COX enzyme, non-aspirin NSAIDs usually do not provide significant long-term inhibition of blood platelet aggregation [10]. 

The classic non-aspirin NSAIDs block both COX-1 and COX-2 isozymes to varying degrees, by binding an arginine molecule at position 120 halfway up their channel, thereby inhibiting access of arachidonic acid to the catalytic site and thus ultimately inhibiting the synthesis of prostaglandins, PGI2, and thromboxanes [22,23]. 

The discovery of the two isoforms of COX by Philip Needleman in 1989 and the subsequent clarification of their 3-dimensional structures provided the rationale for the development of COX-2 selective NSAIDs [5]. An ideal NSAID would selectively inhibit the inducible COX-2 isoform, thereby reducing inflammation and pain, without acting on the constitutive COX-1 isoform, thereby minimizing toxicity. A group of rather bulky NSAIDs was developed, having a rigid side extension that binds within COX-2’s unique side-pocket, thereby being able to access and block COX-2, but not the narrower COX-1 enzyme. The COX-2-selective covalent binding within the COX-2 side-pocket proved to be semi irreversible, thus lastingly inhibiting access of arachidonic acid to the catalytic site [24]. In the 1990s a number of pharmaceutical companies tested and developed this hypothesis and by 1995 the first generation of COX-2-selective NSAIDs, celecoxib (Celebrex®) and rofecoxib (Vioxx®), entered clinical trials, with many other variants eventually being approved for use in the treatment of pain, with rheumatoid arthritis and osteoarthritis being their main indications.

**Figure 1:**
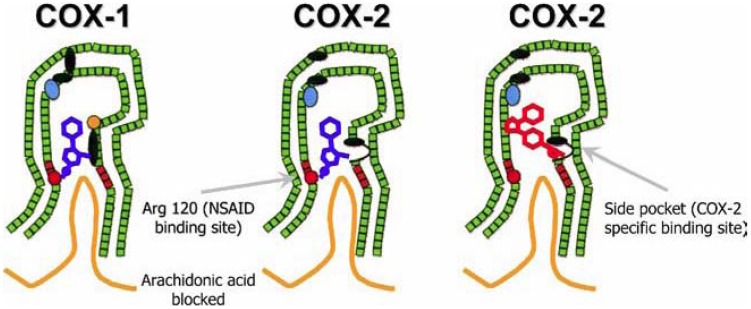
Left: schematic representation of the inhibition of COX-1 (large green figure) by a nonselective NSAID (central blue figure). The entrance channel to COX-1 is blocked by the NSAID. Binding and transformation of arachidonic acid (bottom yellow figure) within COX-1 is prevented. Middle: inhibition of COX-2 by a nonselective NSAID (central blue figure). Right: inhibition of COX-2 by COX-2 selective NSAID (central red figure). The COX-2 side pocket allows specific binding of the COX-2 selective NSAID’s rigid side extension. The entrance channel to COX-2 is blocked. The bulkier COX-2-selective NSAID will not fit into the narrower COX-1 entrance channel, allowing uninhibited access of arachidonic acid into COX-1. Adapted from Hawkey CJ [23].

### 3.3. The Dangers Associated with Pain Relief

It is widely agreed upon that NSAIDs are effective analgesic, antipyretic, and anti-inflammatory drugs, especially in arthritic diseases. However, their use is limited by serious adverse effects. The earliest recognised of these side effects was that NSAIDs share aspirin’s trait of gastro-intestinal toxicity, mediated by the inhibition of COX-1 effects on the gastric mucosa. The spectrum of NSAID-related gastro duodenal toxicity can be categorized into three groups: (I) subjective symptoms like heartburn, dyspepsia, nausea, and abdominal pain are most common, occurring in 15 to 40% of NSAID users and motivating 10% to change or discontinue their NSAID use; (ii) superficial gastro duodenal mucosal lesions such as erosions and asymptomatic ulcers, occurring in 5 to 20% of NSAID users, which may heal spontaneously; (iii) serious gastro duodenal ulcers leading to life-threatening complications like perforation, symptomatic ulcers, and bleeding (perforation, ulcer, bleeding; PUB) occurring in 1% to 2% of chronic NSAID users, with an associated mortality rate of 10% to 15% [10,25,26,27]. Subjective symptoms are poorly correlated with the development of gastro duodenal ulcers. Most NSAID users with subjective symptoms show no endoscopic gastro duodenal damage, while up to 58% of patients who present with life threatening NSAID ulcer complications did not have prodromal symptoms [28]. Risk factors for the development of gastro duodenal ulcers are the same as for aspirin, the risk being higher with higher dosage of NSAIDs [14,15]. Much research has been done on preventive strategies for the development of NSAID related gastro duodenal toxicity. These are either directed at maintaining the integrity of the stomach wall and mucous lining, such as the use of COX-2-selective NSAIDs and the concomitant administration of prostaglandin analogues, or alternatively at inhibiting the secretion of gastric acid, such as concomitant use of histamine H2-receptor antagonists or proton-pump inhibitors (PPI) as described in the section on aspirin [29,30,31,32,33]. Eradication of *H. pylori* in selected patients can further reduce the risk of gastro duodenal damage [34,35]. A detailed review of the effects of gastro protective measures in NSAID associated gastro-intestinal toxicity goes beyond the scope of this article.

In the early 2000s the endovascular functions of the COX enzymes were unravelled. COX enzymes proved to play important parts in thrombogenesis [36]. Activated blood platelets produce COX-1-dependent thromboxane TXA2, which acts as a prothrombotic platelet agonist and vasoconstrictor. Nearby endothelial and smooth muscle cells produce COX-2-dependent prostaglandin I2 (PGI2), especially after cell damage has occurred [37]. PGI2 is an antithrombotic platelet inhibitor and vasodilator and thus modulates the interaction between activated platelets and the endovascular wall. Cell damage, atherosclerotic plaques, and laminar shear forces selectively up-regulate the expression of COX-2 by endothelial cells in an attempt to maintain homoeostasis [38]. Understanding these mechanisms, one could infer that, in clinical syndromes associated with platelet activation, COX inhibition by any NSAID, but especially by COX- 2-selective NSAIDs, may increase the risk for cardiovascular events [37]. As their effect is temporary and reversible, only continuous high dosage of nonselective NSAIDs will considerably inhibit COX-1 and COX-2. However, COX-2-selective NSAIDs may, by their irreversible covalent binding of COX-2, strongly impair the synthesis of endothelium derived antithrombotic and vasodilatory prostacyclin while lacking COX-1-inhibiting effects on platelet aggregation, thus tipping the scales of homeostasis in favour of thrombogenesis and vasoconstriction [37]. In 2004 Merck Sharp and Dohme was prompted to remove its COX-2-selective NSAID rofecoxib (Vioxx®) from the market because of the results of the Adenomatous Polyp PRevention On Vioxx study, showing an 18-month rate of thrombotic events of 1.5 per 100 patient-years with rofecoxib *versus* 0.78 per 100 patient years with placebo (relative risk, 1.92) [38]. Since then many studies have investigated the effects of both COX-2-selective and nonselective NSAIDs on cardiovascular event rates with conflicting results. One meta-analysis assessed the effects of COX-2-selective and nonselective NSAIDs on the risk of vascular events in published and unpublished tabular data from 138 randomized trials that included a comparison of a COX-2-selective NSAID *versus* placebo or a COX-2-selective NSAID *versus* a nonselective NSAID with a treatment duration of at least 4 weeks [39]. Selective COX-2 inhibitors were associated with a moderate increase in the risk of serious vascular events compared with placebo (rate ratio, 1.42), which was chiefly attributable to an increased risk of myocardial infarction (rate ratio, 1.86). High-dose regimens of nonselective NSAIDs were associated with a similar increase in risk of vascular events compared with placebo (rate ratio, 1.51 for ibuprofen, 1.63 for diclofenac), with the exception of high-dose naproxen (rate ratio, 0.92) [39]. Another systematic review and meta-analysis assessed the risks of serious cardiovascular events with individual COX-2- selective and nonselective NSAIDs in 17 case-control studies and six cohort studies [40]. Use of rofecoxib was associated with a significant dose-related relative risk of serious cardiovascular events during the first month of treatment (relative risk, 1.33 with 25 mg or less daily; relative risk, 2.19 with more than 25 mg daily). Celecoxib was not associated with an elevated risk (relative risk, 1.06). Among the nonselective NSAIDs, diclofenac had the highest risk (relative risk, 1.40). For the nonselective NSAIDs ibuprofen (relative risk, 1.07), piroxicam (relative risk, 1.06) and naproxen (relative risk, 0.97), no significant relationship with serious cardiovascular events was found [40]. These results were similar to a meta-analysis assessing the comparative risk of myocardial infarctions with COX-2-selective and nonselective NSAIDs in case-control studies, cohort studies, and randomized controlled trials in colonic adenomas and arthritis, which found an overall small risk of MI with NSAIDs and COX-2-specific drugs, rofecoxib showing the highest risk (relative risk, 1.25 in six cohort studies, 387,983 patient years), possibly due to the long half life of this drug compared to the other compounds. The pooled data of fourteen randomized controlled trials in arthritis with 45,425 patients showed more myocardial infarctions with COX-2-selective NSAIDs (odds ratio, 1.6), but fewer serious upper gastrointestinal events (odds ratio, 0.40) [41]. A last meta-analysis on the cardiovascular risk of celecoxib on the patient-level pooled adjucated data from 7950 patients in six placebo controlled trials comparing celecoxib with placebo for conditions other than arthritis with a planned follow up of at least three years, showed an increase in risk with higher dose regimens, the risk being lowest and nonsignificant for the 400-mg-QD dose (hazard ratio, 1.1) and highest for the 400-mg-BID dose (hazard ratio 3.1) [42]. 

This relationship between dose and cardiovascular risk of NSAIDs that do not completely inhibit COX-1, might be caused by the degree of COX-2 inhibition of the individual compound, as illustrated by the study of García Rodriguez [43]. In this study agents with a degree of COX-2 inhibition <90% at therapeutic concentrations (ibuprofen, meloxicam, celecoxib, and etoricoxib) where associated with an RR of 1.18 (95% CI 1.02–1.38) of MI, compared to an RR of 1.60 (95% CI 1.41–1.81) for agents with a degree of COX-2 inhibition ≥90% (rofecoxib, indomethacine, diclofenac, and piroxicam).

The results of two Danish studies warrant particular caution in prescribing both selective and non-selective NSAIDs in patients with previous myocardial infarction. In the first study on a cohort of 58432 patients discharged after a first-time MI between 1995 and 2002, 9773 experienced rehospitalisation for MI, and 16,573 died. Usage of COX-2 inhibitors in all dosages, and nonselective NSAIDs in high dosages was associated with an increase in mortality, with low NNHs of 13 (95% CI 10–20) for rofecoxib, 14 (95% CI 10–24) for celecoxib, 45 (95% CI 29–102) for ibuprofen, 24 (95% CI 16–45) for diclofenac, and 143 (95% CI 10–20) for other NSAIDs respectively [44]. The second study, conducted between 1997 and 2005, used the same design on two apparently healthy samples of the Danish population. In the sample of 153,465 individuals without any conceivable previous risk factor the NNHs for death of all causes were 14 (95% CI 10–25) for rofecoxib, 20 (95% CI 13–43) for celecoxib, 432 (95% CI 184–1251) for ibuprofen, and 77 (95% CI 51–158) for diclofenac [45].

Based on a review of available data from long-term placebo- and active-controlled clinical NSAID trials, the FDA has concluded that an increased risk of serious adverse cardiovascular events may be a class effect for all NSAIDs, COX-2-selective and nonselective alike (excluding aspirin). Therefore the FDA has requested the package insert for all NSAIDs to be revised and to include a boxed warning highlighting both the presumed increased risk of cardiovascular events as well as the well-described risk of serious, and potentially life-threatening, gastrointestinal bleeding. The FDA has also requested that the package insert for all NSAIDs include a contraindication for use in patients immediately postoperative from coronary artery bypass graft surgery [46]. The EMEA, the European couterpart of the FDA, draws a different conclusion in its statements on COX-2 selective and non-selective NSAIDs from 2005 and 2006. This agency differentiates between the two groups, regarding COX-2 inhibitors contra-indicated in patients with ischemic heart disease or stroke, and cautioning the use of these compounds in patients with risk factors for cardiovascular disease, giving the non-selective NSAIDs the benefit of the doubt [47]. An algorithm for the prescription of NSAIDs in patients based on individual gastro-intestinal and cardiovascular risk profiles, in accordance to the AHA and ACG guidelines, is presented in Table 1 [48,49,50].

**Table 1:** Algorithm for NSAID prescription based on gastro-intestinal (GI) and cardiovascular (CV) risk factors.

	Low GI risk	Moderate GI risk (one or two risk factors)	High GI risk (more than two risk factors)
**Low CV risk**	Non-selective NSAIDs	Non-selective NSAID + PPI or COX-2 + PPI	COX-2 + PPI
**High CV risk**	Naproxen + PPI	Naproxen + PPI	No NSAIDs

GI risk factors include history of ulcers, age over 60 years, high dosage of NSAID, concomitant corticosteroids, anticoagulants, aspirin, platelet inhibitors, and serotonin reuptake inhibitors, *Helicobacter pylori*, diabetes mellitus, heart failure, and rheumatoid arthritis. Proton pump inhibitor (PPI) may also be read as misoprostol 400 to 800 mg. Evaluation of CV risk is according to the judgment of the prescribing physician. Patients with a high CV risk should receive prophylactic low-dose aspirin. If additional NSAID therapy is required, naproxen is the preferred NSAID. Naproxen should be taken 2 hours after aspirin. COX-2: COX-2 selective NSAID. Adapted from ref. [48].

NSAID use has also been associated with the development of hypertension and edema and with exacerbation of pre-existing heart failure. These complications of NSAID use can be explained by NSAID-induced inhibition of the physiologic production of vasodilatory prostaglandin in individuals with an increased activation of the renin-angiotensin and sympathetic nervous system, as is the case in hypertension or states of effective volume depletion, such as heart failure, cirrhosis, and true volume depletion. In these situations NSAID use may induce systemic vasoconstriction by blocking the compensatory release of vasodilatory prostaglandins, causing an increase in afterload and a reduction in cardiac contractility and cardiac output [51]. Depending on the patient’s cardiac reserve, volume status, sodium balance and use of certain antihypertensive medications (with the exception of long acting calcium antagonists), use of NSAIDs can cause sodium and fluid retention, exacerbate heart failure or evoke an elevation in blood pressure averaging 3 to 6 mmHg [52]. Inhibition of renal vasodilatory prostaglandins, which in these situations preserves renal blood flow and glomerular filtration rates by relaxing preglomerular resistance and antagonizing the local vasoconstrictor effects of angiotensin II and norepinephrine, may disrupt a fragile balance and cause reversible renal ischemia, with a subsequent decline in glomerular hydraulic pressure and glomerular filtration rate, leading to acute renal failure [53]. 

One meta-analysis in observational studies and randomised controlled clinical trials to determine the risks of cardiac failure with NSAIDs, showed an increase in the occurrence of cardiac failure by 30-100%, the risk being alike in COX-2-selective and nonselective NSAIDs. However, the absolute risk remains small: less than one patient developed NSAID attributable heart failure per hundred patient years of NSAID treatment. Pre-existing heart failure was associated with the highest risk. Other studies found NSAID use not to be associated with a first occurrence of heart failure, but only with exacerbations of pre-existing disease [54,55,56]. 

The risk of developing hypertension in persons without a previous history of hypertension was investigated in the Nurses’ Health Study II, a prospective study of over 80,000 women of 31 to 50 years of age. In this study the relative risk for the development of hypertension after two years of follow-up was 1.86 with NSAIDs compared with non-NSAIDs, with the exception of aspirin (115). In a recent meta-analysis of 51 randomized clinical trials involving COX-2-selective NSAIDs, with a total of 130,541 participants in whom blood pressure data were available, significantly increased rates of incident hypertension were found in users of COX-2-selective NSAIDs compared to placebo ( risk ratio 1,49) and compared to nonselective NSAIDs (risk ratio 1,12) [57]. The results were mainly driven by rofecoxib (risk ratio 1,87 *vs.* placebo and 1,53 *vs.* nonselective NSAID) and etoricoxib (risk ratio 1,52 *vs.* nonselective NSAID). Comparisons between COX-2-selective NSAIDs and naproxen *versus* COX-2-selective NSAIDs and non-naproxen nonselective NSAIDs showed a higher risk ratio for the development of hypertension (1,31 COX-2 *vs.* naproxen; 1,08 COX-2 *vs.* non-naproxen), but smaller weighted mean differences in systolic and diastolic blood pressure for naproxen *vs.* non-naproxen NSAIDs [57].

In a study on the effect of COX-2 inhibition on renal function in healthy sodium depleted elderly patients that were randomized to rofecoxib 12.5 mg daily, rofecoxib 25 mg daily, indomethacin 50 mg three times daily, or placebo for five days, it was found that glomerular filtration rate was significantly lowered with rofecoxib 12.5 mg (8.4 mL/min lower), rofecoxib 25 mg (7.8 mL/min lower), and indomethacin 150 mg (6.0 mL/min lower) [58]. Another study, with a nested case control design, showed that hospitalization for acute renal failure was correlated with initiation of NSAID use among 121,722 patients older than 65 years of age [59]. The risk of acute renal failure was highest within 30 days of starting treatment and receded thereafter. The relative risk for acute renal failure was comparable among rofecoxib (relative risk, 2.31; 95% CI, 1.73 to 3.08), naproxen (relative risk, 2.42; 95% CI, 1.52 to 3.85), and nonselective, non-naproxen NSAIDs (relative risk, 2.30; 95% CI, 1.60 to 3.32) but was slightly lower with celecoxib (relative risk, 1.54; 95% CI, 1.14 to 2.09). 

Finally, by unknown pathophysiologic mechanisms NSAID use is also associated with renal failure due to acute interstitial nephritis, membranous nephropathy, and minimal change disease nephrotic syndrome. Affected patients typically present with hematuria, pyuria, white cell casts, proteinuria, and acute renal insufficiency. Spontaneous recovery usually occurs within weeks to months after therapy is discontinued [60]. Subsequent administration of NSAIDs should be avoided as relapse may occur with rechallenge.

While the NSAID associated development of hypertension, edema, heart failure and renal insufficiency may occur with COX-2-selective and nonselective NSAIDs alike, one promising new development may offer some perspective. A new class of anti-inflammatory drugs currently under development are the COX-inhibiting nitric oxide (NO) donators (CINODs). CINODs have been designed to provide the anti-inflammatory and analgesic efficacy of NSAIDs but with improved gastrointestinal and cardiovascular safety by coupling a COX-2 selective or nonselective NSAID with a NO-releasing moiety [61]. *In vivo* CINODs demonstrate similar COX inhibition and anti-inflammatory and analgesic properties compared to their reference NSAIDs while their NO release has been shown to improve gastric mucosal blood flow, to promote gastric healing, to inhibit platelet aggregation, to reduce systemic blood pressure and to preserve vascular, cardiac and renal function. One CINOD currently completing phase III trials is naproxcinod, which couples naproxen to a NO-donating moiety. In a randomized, double-blind, 13-week, placebo- and naproxen-controlled trial of 916 patients with osteoarthritis, naproxcinod significantly reduced systolic blood pressure compared to naproxen, especially in hypertensive patients treated with renin-angiotensin blocking agents, with a 6.5 mm Hg difference in mean change from baseline in systolic blood pressure between naproxen and naproxcinod (p < 0.02) [62]. Further trials are awaited. 

### 3.4. Combining Aspirin and NSAIDs: the Devil in Disguise?

Since both aspirin and nonselective NSAIDs bind blood platelets’ COX-1 enzyme, concomitant administration of both drugs may interfere with the beneficial effect of aspirin on the risk of thrombotic cardiovascular events. One study examined the effects of ingestion of 400 mg ibuprofen two hours before or two hours after a regular prophylactic dose of 81 mg aspirin [63]. Serum thromboxane B2 levels and platelet aggregation were maximally inhibited with the administration of aspirin before ibuprofen. In contrast, aspirin’s inhibition of serum thromboxane B2 formation and platelet aggregation was prevented with a single daily dose of ibuprofen before aspirin, as well as when multiple daily doses of ibuprofen were given. The concomitant administration of rofecoxib, acetaminophen, or diclofenac before or after aspirin did not affect platelet inhibition [63]. Similar effects have been described with naproxen in one study, where a single dose of naproxen two hours before aspirin interfered with the antiplatelet effect of aspirin [64]. Nonselective NSAIDs compete with aspirin for a common binding site on the platelet’s COX-1. The presence of a nonselective NSAID at this site prevents aspirin from binding and irreversibly acetylating a serine residue on COX-1 [51,65]. The half life of a specific nonselective NSAID determines the duration of its clinically relevant aspirin blocking effect. Aspirin causes an irreversible and nearly complete blockade of COX at low doses, while the blockade caused by ibuprofen at therapeutic doses is reversible and much less complete, declining rapidly between dosage intervals, reflecting the short half life of the drug [66]. Therefore, one can reason that ibuprofen does not have an inherent platelet aggregation inhibitory effect. Even though naproxen itself has a strong blood platelet aggregation inhibitory effect, the previously mentioned study showed that concomitant use of naproxen also may also give a significant inhibition of the antiplatelet effect of aspirin, albeit smaller than that of ibuprofen. These findings may have strong clinical relevance in patients with cardiovascular disease. Concomitant use of aspirin and ibuprofen or naproxen should be avoided, or at least the NSAID should be administered approximately two hours after aspirin [10]. This pharmacodynamic interaction is not expected in relatively COX-2-preferential (diclofenac) or COX-2 selective NSAIDs.

## 4. Conclusions

Nowadays, one can hardly imagine medicine without aspirin or the nonaspirin NSAIDs, being the cornerstones of modern concepts of cardiovascular event prevention and pain relief. From a pain killing and antipyretic drug, aspirin evolved to a gastric toxin and finally a protector against recurrent thromboembolic cardiovascular events. NSAIDs, being uniquely effective against mild to moderate pain of somatic origin, especially when associated with inflammatory causes, stood their ground despite the emergent reports of several damaging side effects. Development of COX-2-selective NSAIDs significantly reduced the risk of gastrointestinal ulceration, however increased rates of myocardial infarction, heart failure, hypertension and acute renal insufficiency remained. The new class of COX inhibiting nitric oxide donators (CINODs) offers some perspective. The efficacy of both COX-2 selective and nonselective NSAIDs may vary by patient and by indication. In case of inefficacy, substitution by a NSAID from a different chemical class is a reasonable therapeutic option. Nonselective NSAIDs such as ibuprofen or naproxen can interfere with the protective platelet inhibitory effect of aspirin by competitive binding of COX-1. Physicians must take into account both the gastrointestinal and the cardiovascular risks and possible interactions in individual patients when prescribing NSAIDs. One should inform the patient about expected benefits and risks in the consulting-room. The impact of pain and priority of pain relief for patients is shown by a study among Canadian osteoarthritis patients, which showed that most patients were willing to accept some additional risk of ulcer bleeding and to a lesser extent heart attacks or stroke to alleviate their pains [67]. 

As a central dictum in NSAID treatment, physicians should always prescribe the lowest effective dose for the shortest possible time. When starting on NSAIDs gastrointestinal, cardio- and renovascular risks should be estimated. When indicated gastro protective measures should be co prescribed. Patients with a high cardiovascular risk should receive prophylactic low-dose aspirin. If additional NSAID therapy is required, naproxen is the preferred NSAID, in combination with an adequate dose of a PPI or misoprostol, irrespective of the presence of additional gastrointestinal risk factors [48]. Naproxen should be taken two hours after aspirin. COX-2-selective NSAIDs should be avoided in patients with high cardiovascular risk. Patients with both a high cardiovascular risk and a high gastrointestinal risk should avoid NSAID therapy altogether.
